# The Role of PAEHRs in Patient Involvement

**DOI:** 10.1007/s10916-018-1070-y

**Published:** 2018-09-25

**Authors:** Sofie Wass, Vivian Vimarlund

**Affiliations:** 1 International Business School, Jönköping University, P.O. Box 1026, 551 11 Jönköping, Sweden; 2 Department of Computer and Information Science/Human-Centered Systems, Linköping University, 581 83 Linköping, Sweden

**Keywords:** EHR, PAEHR, Patient access, Patient involvement

## Abstract

With increased patient access to data, healthcare services are experiencing change where patients are moving away from being mere passive actors towards becoming more active and involved participants. In this paper, we explore the role of patient accessible electronic health records (PAEHRs) with respect to this increase in patient involvement. The study was performed as a case study and included nine interviews with patients and a survey that was responded to by 56 patients. Our results show that PAEHRs have a role in the enhancement of patient involvement because PAEHRs (i) foster a more balanced relationship between patients and healthcare professionals and (ii) increase access to information.

## Introduction

Modern technology is making healthcare information accessible to patients in ways that were previously not possible [1]. With the more widespread implementation of electronic health records (EHRs), and accompanying legal provisions, patients can now access their electronic health records online [2]. Patient access to data has been identified as an important step towards satisfying the needs of modern healthcare provision and allows for more patient involvement in healthcare [3]. With increased patient access to healthcare data, healthcare services are undergoing a change where patients are moving from being passive actors towards becoming more active and involved participants [4, 5].

At the core of patient involvement lies the role of the patient and the patient’s relationship to healthcare professionals. Historically, the role of the patient was viewed as ‘paternalistic’ in the sense that the patient was considered to be a passive recipient of his or her own treatment [6, 7]. Healthcare is now moving towards engaging with more involved patients. This increase in participation includes (i) involvement in treatment decisions, (ii) involvement in healthcare delivery (including self-care and self-monitoring) and (iii) involvement in development and research [8]. Patient involvement can be influenced by patient-, staff- and organizational factors. Patient factors include helping patients to gain control over their situation and educating patients. Staff can also be trained in how to support patient involvement and to communicate in ways that promote involvement. In addition, organizations can support patient involvement through establishing routines and the use of information technology [8].

Previous studies show that information technology can be used to support patient involvement. Patient accessible electronic health records (PAEHRs) are found to increase patient adherence and compliance [9, 10], patient participation [11] and result in patients feeling more in control of their care [9]. Systems such as OpenNotes have been shown to enhance patient understanding and recall of health information [2, 9, 11], improve trust and communication in the patient-physician relationship [2, 11] and increase patient-centeredness [2]. Some patients have reported privacy concerns, but only a few have claimed that the information caused confusion, worry or offense [9]. In this paper, we focus on patient involvement in the context of treatment decisions with the aim to explore the role of PAEHRs in patient involvement.

## Patient access to electronic health records in Sweden

Since the 1980s, Swedish patients have had the right to request a printed copy of parts of their health record [12]. However, in conjunction with the expansion of electronic health records, online patient access to healthcare information became a topic of discussion. In 2012, Uppsala County Council was the first healthcare provider in Sweden to implement PAEHRs for all of its patients [13], and in February 2015, the Region Jönköping County followed suit and gave all adult patients online access to their electronic health records [14]. PAEHRs are accessible via a national platform for eHealth. This platform enables data integration across different information systems and healthcare actors [15]. Patients can access the information through a secure log-in, using the same electronic ID that they use for banking and other government e-services.

During the present study, the information shown in the PAEHR included medical notes, diagnoses and vaccinations. The medical notes are back-dated to July 1st, 2014. Medical notes that pre-date July 1st, 2014, can be requested and paper copies of these notes will be sent to the patient. Patients can decide to share their electronic health record with other persons, and parents can access their children’s electronic health record until they turn thirteen. At the time of the study, the PAEHR had been implemented for 14 months. Currently, there are differences in the amount of information that the patient is given access to, depending on which region in Sweden that the patient receives healthcare. For instance, patients in Uppsala can access test results, referrals, and even add comments to their medical notes.

## Methods

This study was performed as an exploratory case study and includes data from nine interviews and a survey that was responded to by 56 patients. We also held a workshop with management representatives, so as to gain an understanding of the expected benefits of the PAEHR. The interviews were semi-structured and were used to investigate how patients experienced the PAEHR. The results of the interviews also informed the construction of the survey that was later administered. The interviewees were initially contacted at the county hospital in the region and were later contacted by email to arrange a time to conduct the interview, at the hospital. Four of the respondents were women and five were men, aged between 34 and 83. All of the patients had previously accessed their PAEHR by the time that they were interviewed. The interviews were recorded and subsequently transcribed verbatim. The first author reviewed the transcripts of the interviews and analysed them by using inductive content analysis [16]. We focused on sentences or paragraphs that described the perceived benefits of accessing the PAEHR. These sentences were shortened and labelled with a code. Finally, the codes were compared and discussed by the two authors and unified into themes (Table 2).

The themes that were identified in the transcriptions of the interviews served as a basis for the survey questions. The survey was reviewed by five patients to clarify the questions, and it was revised according to the feedback that was received. The survey was then distributed to patients at three different sites in the region: a primary care unit and two outpatient clinics. The care unit and outpatient clinics were selected as sites where the survey was distributed in an attempt to reach patients who were currently in contact with the healthcare system. The survey was paper-based and was given to patients by a medical secretary during their registration for their visit. A letter accompanied the survey, stating that participation was voluntary and anonymous. Only patients who had used the PAEHR were eligible to take part in the study. The completed survey could be deposited in a box at the reception or sent by mail to the researchers. The distribution of surveys lasted for two weeks in May 2016, and because it was anonymous, no reminders were sent. In total, 56 patients completed the survey (12 patients declined to take part in the study and 24 did not return the survey).

The survey included questions regarding the use of the service, statements about information access, patient involvement and patient-professional communication. With respect to the statements that focused on attitudes, we examined the results across a 5-level grade, including the responses “agree”, “somewhat agree”, “neither agree nor disagree”, “somewhat disagree” and “disagree”. The percentage of patients who “agreed” or “disagreed” with the statements was calculated for each question. The response rate for the survey was 61% (*n* = 56). 70% were men, between 35 and 83 years of age, and 68% used the Internet several times a day.

## Results

### Value of the PAEHR

Almost all the patients viewed the PAEHR as a good or very good initiative (94%, *n* = 56) and none of the patients considered it to be a negative initiative. The respondents were asked to choose the words they considered most suitable to describe the service out of a list of 23 ‘positive’ and ‘negative’ expressions (Table 1).

**Table 1:** The choice patients made when presented with a selection of expressions (*n* = 45)

Rank	Expression	% (n)	Rank	Expression	% (n)
1	trustworthy service	70% (31)	8	openness towards citizens	44% (20)
2	time saving	64% (29)	9	enables participation	44% (20)
3	a citizen’s right	62% (28)	10	enables responsibility	42% (19)
4	easily accessible information	60% (27)	11	the patient can influence	33% (15)
5	easy to navigate	51% (23)	12	difficult to navigate	7% (3)
6	secure information management	49% (22)	13	undeveloped service	4% (2)
7	rich in information	47% (21)	14	creates misunderstandings	2% (1)

The results show that patients almost exclusively chose positive expressions. The most common expressions were: trustworthy service, time saving, a citizen’s right and easily accessible information. Similar patterns were found in the interviews, where the patients described different benefits with the PAEHR (Table 2).

**Table 2:** Themes focusing on the benefits of the PAEHRs, as identified from the interviews

The service…	Number of respondents
help*s* me to ensure that we reached a mutual understanding	7
help*s* me to ensure that I understand what the healthcare professional said	6
improves the access to health information	6
makes it possible to assist relatives	6
increases patient involvement	5
is a more secure way to send private information	2

For instance, the patients described the online process to be more secure than sending information by mail: *“Since the patient portal requires a secure log in there is no risk that any external [person] will read it by mistake. On the other hand, someone can steal and open a letter in the mailbox. I feel more secure if I get the response that way.”* Several patients also described the information as easily accessible and that the PAEHR saves time. *“It helps a lot, it is easily accessible, the communication is faster and you get a quicker response to how you experienced the care this time, and what they said.”* The improved access to information was confirmed by the survey, where almost all patients agreed or somewhat agreed that it was easier to access information (96%, Table 3).

**Table 3:** Perceptions of patient involvement

The service makes it easier to… /for me…	Agree or somewhat agree	Neither agree nor disagree	Disagree or somewhat disagree
access information from the appointment/phone call (*n* = 55)	96% (53)	4% (2)	–
more involved in my treatment and/or rehabilitation (n = 55)	81% (45)	16% (9)	2% (1)
understand what was said during the appointment/phone call (n = 55)	89% (49)	11% (6)	–
talk to physicians, nurses, or another person about my situation (n = 55)	82% (45)	16% (9)	2% (1)
discuss what is documented about me (n = 56)	72% (40)	27% (15)	2% (1)
coordinate to ensure that which was talked about was actually documented (n = 56)	80% (45)	18% (10)	2% (1)
take more responsibility for my care (n = 55)	76% (42)	22% (12)	2% (1)
affect my care more actively (n = 55)	62% (34)	36% (20)	2% (1)

### The PAEHR and patient involvement

The results of the interviews and the survey indicated that patients feel more involved when they can access their PAEHRs online. The survey results showed that 81% of the respondents agreed or somewhat agreed that the service has made them more involved in their treatment (Table 3).

One interview respondent felt more involved due to being provided the opportunity to obtain a proper understanding of the treatment process: *“I can follow the process, what has happened and everything, so of course I feel more involved in the work that they have done with me”.* Another respondent reported: *“You can get a better insight, and actually maybe take another responsibility because you can take part of the information without needing to make a call. You get better knowledge and you become the owner of the question.”*

Another theme that emerged from the interviews dealt with the patients’ understanding of the information that healthcare professional gave them. *“The confirmation, that is important to me, because it can be difficult to follow the information and then, it goes so fast… It was a strength to be able to read in peace and quiet.”* This reported increase in understanding was confirmed in the survey, where 89% of the respondents agreed or somewhat agreed with the statement about understanding what was said. Several interview respondents also mentioned the fact that they could now ensure that they had reached a mutual understanding with the healthcare professional. One respondent used the PAEHR to check if something needed to be discussed during the next meeting. *“I log in after each visit to read and to clarify and to see if there is something specific that I need to talk to the doctor about the next time.”* An improvement in the quality of communication was also indicated by the survey results; 82% of the patients agreed or somewhat agreed that the service made it easier to talk to healthcare professionals about their situation. In addition, 72% agreed or somewhat agreed that it was easier to discuss what was documented about them. 80% of the respondents claimed that it was easier to check whether that which was talked about was actually documented (Table 3).

The survey results showed that 76% of the respondents agreed or somewhat agreed that the service made it easier for them to take responsibility for their care, and 62% stated that it was easier for them to more actively affect their own care. In the interviews, the respondents also mentioned that it was possible for them to assist relatives when given access to their PAEHRs. One respondent had shared it with their children *“It is really good, I can share it with my kids that do not live nearby.”* Another respondent had used it to assist her mother to read the information, *“Then we could log in to her EHR so she could read… It improves the communication.”*

During the interviews, the respondents stated that they were eager to get access to *more* information in the PAEHR and that this would increase patient involvement. One respondent said that: *“When I log in, then I cannot find referrals for instance, I cannot find the test results, the diagnoses are there if you access them and read them but there is still a lot that is missing…when you know that the possibility exists. It will most likely make you even more involved.”* The desire for more information was confirmed in the survey, where the respondents were asked to judge between different types of information that could be added to the PAEHR. The top three additional pieces of information that were requested included test results (85%), access to referrals (61%) and information on the interactions between different drugs (46%) (Table 4).

**Table 4:** Ranking of requested additional PAEHR features that could increase involvement

Features selected in the top three in the survey (*n* = 46).	% (n)	Number of respondents who mentioned the feature in the interviews
Test results	85% (39)	7
Referrals	61% (28)	6
Interactions between different drugs	46% (21)	1
Medical records dated before 2014	35% (16)	2
Reminders about new information in the EHR by SMS or e-mail	22% (10)	1
The ability to report errors in the EHR	15% (7)	1
Information on when to update vaccinations	13% (6)	1

## Discussion

The results show that patients consider the PAEHR to be a good initiative that provides patients with easily accessible information that is time-saving. It is also considered to be a secure way of giving patients access to their own health information. Although there has been previous discussion regarding issues of security and privacy with respect to electronic health records [17, 18], our study does not report on any patient concerns about these matters.

Both the interviews and the survey results show that the PAEHR plays a significant role in patient involvement. In our study, the patients report that the PAEHR increases patient involvement and enhances their understanding of what was said during their medical appointments. The PAEHR confirms their understanding of their treatment, reminds them of what was said and thus complements the meeting between the care provider and patient. These results are consistent with previous studies which have identified various benefits such as a greater understanding of healthcare plans [9] and medical notes that confirm the patient’s understanding of the information that has been provided to them [2, 19].

According to the European Commission [20], one important element of patient involvement is the creation of a more balanced relationship between patients and healthcare professionals. Our results indicate that the PAEHR improves the patient-professional relationship because patients feel that it is easier (i) to reach a mutual understanding of what takes place during the consultation and (ii) to communicate with healthcare professionals about their medical condition and life situation. For instance, the PAEHR was used by patients to ensure for themselves that they were in agreement with what was said during the consultation and to record whether something needed to be clarified during the next appointment.

Based on the survey results, the PAEHR enhances responsibility-taking. Some patients felt that they could affect their care more actively because of the system. However, this point was not identified as a distinct theme in the interviews. Even if patients do feel more involved in their treatment, the survey results and the interviews show that patients wished to access more information than just medical notes, vaccinations and diagnoses. Their wishes included information about test results, referrals and interactions between different drugs. Some of this information is already available to patients in other regions in Sweden [21], and it is important to provide such information on equal terms to all citizens. In fact, the results show that several respondents view the PAEHR as a citizen’s right.

Even if the PAEHR enhances patient involvement, other factors can also be relevant to increase patient involvement. It is thus important to combine the PAEHR with support in how healthcare staff can communicate with their patients in ways that promote patient involvement [8] and encourage patients to access the PAEHR.

## Conclusion

The PAEHR presents a new situation where patients experience their health information as being more accessible than before. The online access to information saves time, increases patient involvement and improves the patient-professional relationship. PAEHRs and similar technologies [8] have an important role in enhancing patient involvement because they foster a more balanced relationship between patients and healthcare professionals. With an improved patient-professional relationship, where patients experience that it is easier to communicate about their medical condition and to reach a mutual understanding, there is a potential to also improve the provided care.

However, it is desirable that even more information from the electronic health record be shared with patients. This includes information about test results, the opportunity to monitor referrals and additional services, such as information on drug interactions. This increased information sharing with patients appears to support a change in the role of the patient, from that of a passive patient to becoming an informed and engaged consumer of healthcare services. Based on the results from this study, it seems desirable to aim for a more widespread use of PAEHRs.

Although this study was performed as an explorative case study, we believe that our results provide some insight into the important area of PAEHRs [22]. Previous studies on PAEHRs have primarily focused on the experiences of patients in certain clinical groups and patients with chronic diseases [5]. This study contributes to the identification of the experience of patients outside these specific groups by focusing on primary care and outpatient units. While this study explored the role of PAEHRs in patient involvement, we have not focused on issues related to the negative aspects of PAEHR and it should be acknowledged that another focus could have highlighted concerns with PAEHRs.

Future studies should not only explore how online access to health data impacts a patient’s healthcare, but also the role of patient-generated data and how it can contribute to the provision of healthcare. Adler-Milstein et al. [3] mention, for instance, how data generated by Fitbits and Apple Watches can be connected to electronic health records. It is thus of interest to examine how self-care and self-monitoring applications and devices might provide important additional information to the electronic health record.
